# Quantification of perineural satellitosis in pretreatment glioblastoma with structural MRI and a diffusion tensor imaging template

**DOI:** 10.1093/noajnl/vdad168

**Published:** 2023-12-19

**Authors:** Rik van den Elshout, Benthe Ariëns, Joost Blaauboer, Frederick J A Meijer, Anja G van der Kolk, Morteza Esmaeili, Tom W J Scheenen, Dylan J H A Henssen

**Affiliations:** Department of Medical Imaging, Radboud University Medical Center, Nijmegen, The Netherlands; Department of Medical Imaging, Radboud University Medical Center, Nijmegen, The Netherlands; Department of Medical Imaging, Radboud University Medical Center, Nijmegen, The Netherlands; Department of Medical Imaging, Radboud University Medical Center, Nijmegen, The Netherlands; Department of Medical Imaging, Radboud University Medical Center, Nijmegen, The Netherlands; Department of Diagnostic Imaging, Akershus University Hospital, Lørenskog, Norway; Department of Electrical Engineering and Computer Science, University of Stavanger, Stavanger, Norway; Department of Medical Imaging, Radboud University Medical Center, Nijmegen, The Netherlands; Department of Medical Imaging, Radboud University Medical Center, Nijmegen, The Netherlands

**Keywords:** brain, glioblastoma (GBM), magnetic resonance imaging, biomarkers, DTI atlas

## Abstract

**Background:**

Survival outcomes for glioblastoma (GBM) patients remain unfavorable, and tumor recurrence is often observed. Understanding the radiological growth patterns of GBM could aid in improving outcomes. This study aimed to examine the relationship between contrast-enhancing tumor growth direction and white matter, using an image registration and deformation strategy.

**Methods:**

In GBM patients 2 pretreatment scans (diagnostic and neuronavigation) were gathered retrospectively, and coregistered to a template and diffusion tensor imaging (DTI) atlas. The GBM lesions were segmented and coregistered to the same space. Growth vectors were derived and divided into vector populations parallel (Φ = 0–20°) and perpendicular (Φ = 70–90°) to white matter. To test for statistical significance between parallel and perpendicular groups, a paired samples Student’s *t*-test was performed. O^6^-methylguanine-DNA methyltransferase (MGMT) methylation status and its correlation to growth rate were also tested using a one-way ANOVA test.

**Results:**

For 78 GBM patients (mean age 61 years ± 13 SD, 32 men), the included GBM lesions showed a predominant preference for perineural satellitosis (*P* < .001), with a mean percentile growth of 30.8% (95% CI: 29.6–32.0%) parallel (0° < |Φ| < 20°) to white matter. Perpendicular tumor growth with respect to white matter microstructure (70° < |Φ| < 90°) showed to be 22.7% (95% CI: 21.3–24.1%) of total tumor growth direction.

**Conclusions:**

The presented strategy showed that tumor growth direction in pretreatment GBM patients correlated with white matter architecture. Future studies with patient-specific DTI data are required to verify the accuracy of this method prospectively to identify its usefulness as a clinical metric in pre and posttreatment settings.

**Key Points**

- Growth of glioblastoma can be quantified and derived from a DTI template using our pipeline.
- Growth vector populations show that visible tumor growth direction is mostly along the white matter.
- Quantifying growth helps in understanding growth patterns due to tumor microenvironment.

**Importance of the Study**

Quantifying radiological structural imaging of contrast-enhancing GBM growth helps in understanding growth patterns and to establish baseline tumor size. In turn, this in vivo study visualizes tumor growth direction in vector bins, corroborating histopathological evidence for perineural satellitosis as described by Scherer. The image registration and deformation strategy could be used in future studies for posttreatment follow-up of GBM patients, hypothetically differentiating between tumor progression and treatment-related abnormalities and potentially aiding in treatment planning, patient management, and outcome.

Glioblastoma (GBM) is the most common malignant brain tumor, comprising roughly half of all malignant brain tumors.^1^ The diagnosis is made by histopathological assessment of tissue obtained via resection or biopsy. On MRI, GBM is seen as a contrast-enhancing lesion with central necrosis surrounded by *T*_2_/FLAIR hyperintense regions representing a combination of perilesional edema and infiltrating tumor cells.^2^ Despite extensive research on optimizing treatment strategies, patient outcome is unfavorable with a median survival of 15 months.^3^ In posttreatment settings, tumor recurrence is observed very often. The limited overall survival and high prevalence of tumor recurrence can be attributed to the infiltrative growth pattern of GBM. GBM tend to grow along white matter tracts and blood vessels, co-opting on nutrients and growing along the path of least resistance.^4,5^ This infiltrative growth exists beyond the contrast-enhancing rim of the tumor and spreads into a large area surrounding the lesion. Since both tumor tissue and tumor-associated edema appear *T*_2_/FLAIR hyperintense, defining the true extent of tumor cell infiltration is almost impossible using conventional MRI.

The co-optive growth pattern of GBM, also known as perineural and perivascular satellitosis, is well-known from microscopy studies and first described by Scherer in 1938.^4^ Perineural satellitosis is characterized by invasion of the area surrounding neurons and perivascular satellitosis by infiltration of Virchow–Robin spaces surrounding blood vessels by neoplastic glial cells.^6^

Advanced imaging modalities have been suggested to better understand the tumor microenvironment and follow-up of GBM.^7^ One such suggestion concerns diffusion-weighted imaging (DWI).^8^ DWI is a noninvasive imaging technique that measures the random displacement of water molecules (i.e. the Brownian motion of water) and its restrictions by compartmentalization in biological tissues. It thereby provides information on the microstructural organization of tissues, and disturbances in this structural organization. In addition, DWI can quantify the directionality of water diffusion using diffusion tensor imaging (DTI).^9,10^ In GBM, the normal organization of the brain is influenced by the presence of tumor cells and therefore, DWI and DTI parameters can change due to GBM infiltration patterns. However, due to the complex interplay between tumor cells and their microenvironment, modeling and quantifying the direction of this co-optive growth pattern in imaging studies remains challenging.^7,11–13^ An important biomarker in determining prognosis and reaction to treatment is the O^6^-methylguanine-DNA methyltransferase (*MGMT*) gene, where hypermethylation of said gene indicates a more favorable outcome.^14^ Previous literature suggests methylation status of the *MGMT* gene has no influence on the GBM growth pattern.^15^

Tumor-associated edema is another factor complicating the successful quantification of GBM infiltration. Studies are therefore focusing on the visible part of tumor and finding ways to use available information for understanding patterns of spread. A recent study quantifying growth of the contrast-enhancing rim of GBM with highly active and vital tumor tissue, through the use of a DTI atlas,^16^ showed a statistically significant portion of the growth parallel to the white matter structures. The ability to identify and quantify the growth pattern of contrast-enhancing tumor tissue along white matter structures could potentially be used in treatment planning and follow-up of GBM patients. Understanding tumor growth may aid in discerning sites of recurrence versus treatment-related abnormalities such as pseudoprogression and radiation necrosis. Concurrent loss of function can be linked to the site of recurrence or pseudoprogression, adding to improved patient management and could also aid with radiotherapy target volume delineation.

By replicating previous DTI studies to quantify GBM growth patterns using an improved image registration and deformation strategy, the aims of this study were to examine the relationship between contrast-enhancing tumor growth direction in GBM and white matter microstructure and *MGMT* status, to determine tumor location preference and to lay the foundation for future studies in posttreatment GBM.

## Materials and Methods

### Ethical Approval

This study was approved by our institutional review board and was registered under file number 2020-6480. Due to its retrospective nature, written informed consent was not required for this study. At our institution, patients may opt-in to share their clinical data for future scientific purposes, providing consent to share their (pseudonymized) data from the time of the start of their treatment.

### Study Population

In this retrospective study, patients with the histopathological diagnosis of GBM who were treated between May 2016 and October 2021 were eligible for inclusion. Patient data was retrieved from the Picture Archiving and Communication System. Patients were included if they had undergone 2 pretreatment MRI examinations: the first one at the time of radiological diagnosis (hereafter termed “diagnostic MRI scan”) and the second one 1 day prior to resection to aid in surgical planning (neuronavigation scan). Both MRI investigations needed to contain a postcontrast *T*_1_-weighted MRI sequence and be carried out with a time interval of at least 14 days in order to accurately detect growth. Patients were excluded if: (1) they were under 18 years of age, (2) they opted out of the data sharing system at our institution and did not allow their data to be used for research, (3) they had a history of neuro-oncological disease or previous neurosurgical treatment, (4) their MRI scans were unavailable, or (5) the quality of their MRI scans was suboptimal due to differences in image quality or spatial resolution between the 2-time points, which could affect the accuracy of deformation field estimation.

### MR Examination

Diagnostic MRI data was acquired on 1.5T or 3T scanners from different academic hospitals and district hospitals in The Netherlands using various unknown imaging protocols. For all patients, only the postcontrast 3D *T*_1_-weighted MRI scans were extracted from the 2 pretreatment imaging examinations. The neuronavigation scan was always performed at the same institution, see Table 2 for details on the acquisition protocol. Scans were performed using gadoteric acid (Dotarem, Guerbet) as a contrast agent. The exact amount of gadoteric acid administered differed per individual, depending on the protocol and the weight of the patient. The neuronavigation scan was performed 10 min after i.v. administration of 0.2 mL/kg (max 15 mL) Dotarem. All scans were assessed clinically according to protocol by a 2-person team of a junior radiologist in training and a supervising senior radiologist. Prior to supplying data to the investigator, all patient data were pseudonymized.

### Glioblastoma Segmentation

The GBM lesions were segmented using a combination of manual and semiautomatic methods with the ITK-snap software, which is specifically designed for 3D medical image segmentation using active contour methods, manual delineation, and image navigation.^17^ The segmentation of the tumor comprised the visible tumor region, encompassing both the necrotic center and the contrast-enhancing rim. Two investigators (R.v.d.E. and J.B.; <3 years of experience with neuroimaging) performed the segmentations independently, and any discrepancies were manually corrected. To ensure the accuracy of the segmentations, a third researcher oversaw the segmentation process (D.H.; a resident radiologist with over 8 years of experience with experimental and clinical neuroimaging).

### Image Registration Steps

**Figure 1.**
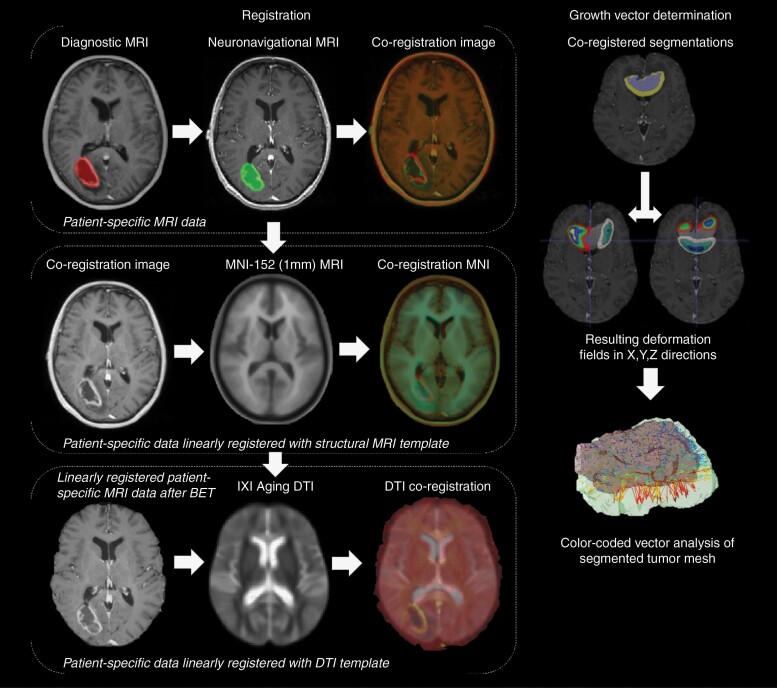
Overview of the image registration and tumor growth vector deformation field resulting in the vector populations parallel or perpendicular to white matter according to the DTI atlas. Diagnostic and neuronavigational scans coregistered to MNI space, segmented tumor deformation field in *XYZ* directions calculated. DTI template coregistered to MNI, deformation field put along white matter orientation leading to vector bins.

A simplified overview of the registration and deformation steps can be found in Figure 1. First, the postcontrast *T*_1_-weighted MRI scans from the 2-time points were coregistered for each patient by translating the diagnostic MRI scan to the neuronavigation MRI scan; the resulting transformation matrix from the affine registration was then applied to the tumor segmentation mask derived from the diagnostic MRI scan. Then, all MRI data were coregistered to the MNI152 1 mm brain template, which is a mean derivative from 152 structural MRI scans of healthy individuals with an isotropic voxel size of 1 mm and produced by the Montreal Neurological Institute (MNI).^18^ Coregistration was performed with 12-point linear affine registration using the FLIRT algorithm from the FMRIB Software Library (FSL).^19^ Thereafter, the linearly transformed patient-specific postcontrast *T*_1_-weighted images were skull-stripped using the Brain Extraction Tool of FSL.^20^

To estimate the correlation of tumor growth directions with white matter structures, we employed the IXI Aging DTI template as a directional reference standard; this template was constructed using a cohort of 51 normal elderly subjects (age range: 65–83 years; 21 males and 30 females) selected from the IXI brain database, which was developed collaboratively by Imperial College of Science Technology & Medicine and University College London.^21^ More information on the IXI Aging DTI template can be found elsewhere.^21–23^ The IXI aging DTI template consisted of tensor intensities and vector information.^23^ The Dxx, Dyy, and Dzz tensors were extracted from the template using an in-house written code using MATLAB R2022b. The obtained Dxx, Dyy, and Dzz files of the DTI template were registered with the linearly transformed skull-stripped postcontrast *T*_1_-weighted data of each patient using the advanced neuroimaging tool (ANTs) script packages.^24,25^ The skull-stripped *T*_1_-weighted neuronavigation scan was used as the fixed image for the coregistration of the DTI and MR images. Furthermore, the N4 bias correction tool, available in ANT packages, were implemented to minimize field inhomogeneity effects through parametric bias field correction and nonuniform intensity normalization.^25,26^ Registration was performed using the affine function since the white matter in the DT images should be deformed by applying a deformable registration method. The quality of all registrations was visually inspected and verified by one of the investigators (R.v.d.E.) using Synchroview in Mevislab.^27^

### Deformation Field Estimation

The tumor regions on the *T*_1_-weighted scans at the 2-time points were masked out; then, the masked-out tumor region on the diagnostic MRI scan was registered to the masked-out tumor region on the neuronavigational MRI scan using the nonlinear function in ANTs, which is based on the b-spline function. This resulted in a localized multicomponent deformation field, which characterized the changes in tumor shape and volume between the 2 MRI time points. The deformation field was converted into the *x*, *y* and *z* vectors with the ITK-SNAP tool Convert3D. Using in-house coded software (MATLAB R2022b), the obtained deformation field and the DTI vectors were used to calculate the voxel-wise alignment between tumor growth in each patient and white matter microstructure on the IXI DTI template. Tumor growth direction and DTI-derived white matter microstructure orientation were separated into 9 bins of 10°, covering the full range from 0° to 90° magnitude. Certain thresholds needed to be defined to investigate the orientation of tumor growth in relation to white matter microstructure. Following the approach taken by Esmaeili et al.,^16^ a threshold was established for defining the tumor growth direction as “parallel” to the white matter microstructure when the vector populations (Φ) ranged between 0° and 20°. Conversely, vector populations (Φ) ranging between 70° and 90° were defined as indicating a perpendicular tumor growth direction with respect to the orientation of the white matter microstructure.

### Statistical Analyses

The tumor locations and volumes of all included patients coregistered into the MNI space were superimposed to construct a so-called tumor frequency map. The frequency of tumor location was color-coded blue to red representing low to high occurrence, respectively, to show tumor location preference and spatial overlap between patients. Statistical analyses were performed using IBM SPSS software (version 27; IBM Corp.). Demographic data and tumor growth direction vector populations were tested for normality using the Kolmogorov–Smirnov test. Demographic data were represented as mean ± SD if normally distributed, or as a median with range (minimum–maximum) if not normally distributed. To test for statistical significance between the parallel and perpendicular vector populations, a paired samples Student’s *t*-test was performed. Statistical significance was set at *P *≤ .05.

## Results

### Population

**Figure 2.**
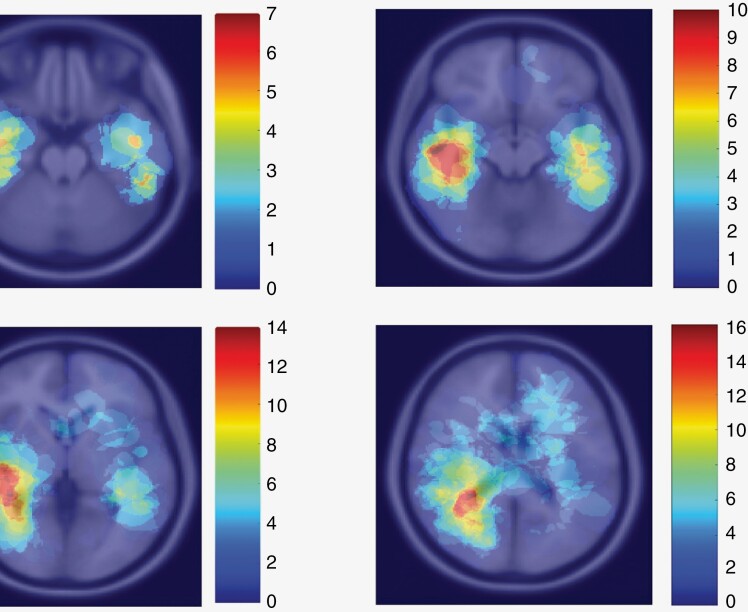
Tumor location frequency map on the MNI atlas, the blue color corresponds to fewer tumors localized, while the red color depicts high tumor occurrence. Tumor occurrence depicted is relative to the second MRI time point. These slices were selected at regular intervals to give a general idea of tumor localization.

Seventy-eight patients met the inclusion criteria. An overview of the study population can be found in Table 1. In Figure 2, the tumor frequency map is depicted. Although this dataset shows a predisposition for right-sided temporoparietal localization, intra-axial tumors can occur practically anywhere, with a predisposition for the frontal lobe.^28^

**Table 1.** Population Data

	Male	Female	Total
Count	32	46	78
Mean age	61.2 (13.2)	61.8 (12.8)	61.4 (12.9)
MGMT hypermethylated/unmethylated/unknown	11/9/12	24/7/15	35/16/27
Isocitrate dehydrogenase (IDH) wildtype	32	46	78
Median time between scans (minimum–maximum)	19 days (6–72 days)	20 days (4–97 days)	19 days (4–97 days)

**Table 2.** Neuronavigation Scanning Protocol

Parameter	*T*_1_-MPRAGE
Repetition time (TR) (ms)	2200
Echo time (TE) (ms)	2.97
Slice thickness (mm)	1
Matrix size (pixels)	256 × 256
Resolution (mm × mm)	1 × 1
Acquisition plane	Transversal
Acquisition time (min)	05:30

### Vector Populations

**Figure 3.**
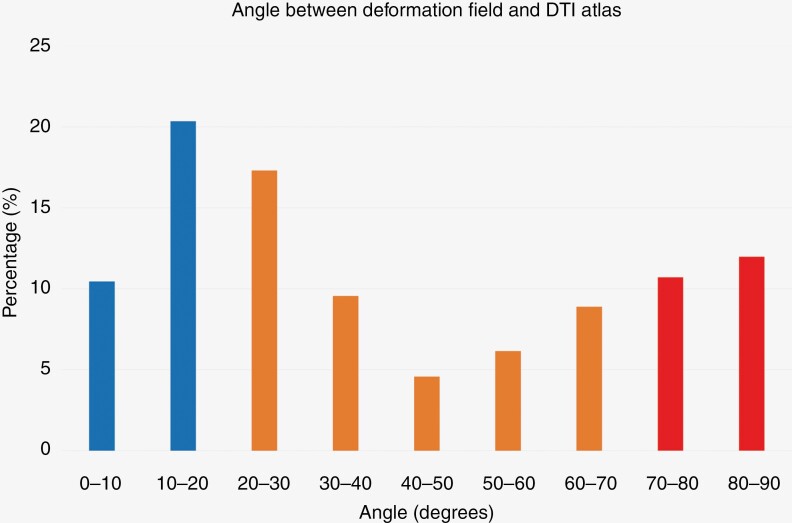
Angle distribution between vector bins of the DTI template and obtained deformation field. Vector populations parallel to white matter (0-20 degrees) occur more often than vector populations perpendicular to white matter (70-90 degrees). Middle vector populations (20-70 degrees) represent the vector groups between parallel and perpendicular.

A one-sample Kolmogorov–Smirnov test showed that the mean tumor growth direction classified as occurring within vector populations |Φ| < 20° (parallel) and |Φ| > 70° (perpendicular) was distributed normally ((D(87) = 0.038; *P* = .20) and (D(87) = 0.043; *P* = .20), respectively). Figure 3 shows a histogram analysis of the binned tumor growth directionality within this cohort per vector population. The included GBM lesions showed a predominant preference for growth direction along DTI-derived white matter microstructure orientation, with a mean percentile growth of 30.8% (95% CI: 29.632.0%) in line (0° < |Φ| < 20°) with white matter direction. Within this category, a 10.4% (95% CI: 9.9–11.0%) evolution of tumor growth direction over time occurred within the 0–10° vector population. The tumor growth direction classified as perpendicular growth with respect to white matter microstructure (70° < |Φ| < 90°) showed to be 22.7% (95% CI: 21.3–24.1%) of total tumor growth direction over time. Within this category, 12% (95% CI: 11.1–12.9%) of the tumor growth direction over time was classified as occurring within the 80–90° vector population. The paired samples *t*-test showed statistical significance between parallel and perpendicular populations (*P* < .001, Cohen’s *d* 0.74), implying that tumor growth parallel to white matter tracts is significantly more common than a perpendicular increase. There was no significant difference between MGMT methylation status and tumor growth rate (*P* = .53), or degree of parallel growth (*P* = .274) or perpendicular growth (*P* = .16) using the one-way ANOVA test.

## Discussion

The objective of this study was to examine the relationship between contrast-enhancing tumor growth direction in GBM and white matter microstructure. Our results show that GBM lesions on MRI have preferential growth along the course of white matter tracts, as opposed to perpendicular to their orientation. Visualizing this growth pattern on MRI data is an important and relatively novel observation as it sheds light on the complex interplay between tumor growth and white matter structure. The results corroborate the growth patterns and perineural satellitosis of GBM as described in histopathological studies^4^ and suggest that the contrast-enhancing part of GBM follows the infiltrative satellitosis. This, in turn, leads to hypoxia- and inflammation-induced contrast enhancement as described in the literature.^29,30^ To explain this phenomenon, various studies suggested a complex interplay between GBM cells and the neural microenvironment, connecting the involvement of extracellular matrix proteins to the affinity of GBM cells with myelinated axons. An extensive review of the molecular mechanisms underpinning this growth pattern, however, lies beyond the scope of this manuscript and is given elsewhere.^31–33^ What stands out is the presence of a second maximum in the 80–90° vector group, with a minimum in the 40–50 vector group. It is, as of yet, unclear whether this phenomenon can be attributed to biological or anatomical influences or could potentially be a mathematical artifact due to methodology. Apart from directional growth along white matter tracts, diffuse gliomas tend to grow along (micro)vasculature as well. Blood vessels, containing a basement membrane, provide migrating GBM cells a structure to grow along. This component of GBM growth could not be included in this paper; however, its mechanism could explain the variation in growth directionality that we observed. It should also be noted that the *T*_1_-contrast-enhancement area used for this study is smaller than the enhanced signal on FLAIR/T2w.^34^ As the FLAIR signal also represents edema and neuroinflammation, however, this is also not an accurate depiction of the infiltrative tumor field. So, the ground truth lies somewhere in between, with no known biomarkers to accurately detect total tumor infiltration. MGMT status did not influence growth rate or directionality in the pretreatment setting of our studied population, corroborating current literature findings on phenotypical tumor biology.^15^ Additionally, it was observed that the patients included in the study exhibited a distinct tumor occurrence location in the temporoparietal lobe, deviating from the previously documented descriptions in the existing literature, and exhibiting a preference for frontal lobe incidence. However, it is worth noting that there is evidence of a partial agreement, as a subset of patients exhibited tumor overlap between lobes.^28,35^

Studies on mathematical modeling of GBM growth using DTI have primarily focused on predicting sites of invasion in accordance with white matter architecture and remain highly fundamental, relying mainly on simulations and thereby lacking clinical implementation.^11–13^ The overarching goal of these studies was to generate mathematical models of GBM growth to visualize the growth pattern known from histological studies. Partial differential equations incorporate more advanced tumor growth models and aim to predict the direction of growth and/or recurrence.^36^ Further research is needed to assess the clinical value of these mathematical models. The current study uses clinical imaging data in order to assess growth retrospectively, providing a stepping stone for clinical implementation and possibly for verification of the aforementioned models.

A significant methodological limitation of this study comprises the lack of patient-specific DTI data to quantify the growth directions. Although advanced image registration and deformation strategies were used, the deformation and destruction of white matter through mass effect and invasive growth of the tumor could not be entirely corrected for, leading to the displacement of fibers from their anatomically correct orientation in the template and cannot be accurately captured by any image coregistration, be it rigid or deformable, making this approach a crude approximation of the actual patients their white matter microstructure. Another limitation of this study is the lack of histopathological correlation. Ideally, these results would be verified postmortem on 1 or 2 subjects to accurately determine the feasibility of the current strategy. However, as the results of this study corroborate the findings of other DTI studies and results from histopathological studies, the impact of these methodological limitations seems to be finite. Nevertheless, a future study using patient-specific DTI data could help further elucidate the precision of quantifying tumor growth direction in relation to white matter microstructure. Additionally, the main spatial directions of the brain, being mediolateral, rostrocaudal, and dorsoventral directions, define the directions the major white matter tracts follow by approximation in every brain. This could be used as a general model to study the orientation of the deformation vectors as well, providing a crude approximation of the deformation in line with or perpendicular to those large white matter tracts. And may also be a future direction in order to validate the hypotheses and results stated here.

Future perspectives for this method of quantifying tumor direction growth in GBM could entail the posttreatment surveillance of GBM patients. Distinguishing tumor progression from treatment-related abnormalities currently poses a significant challenge in neuro-oncological radiology, particularly in patients who have received focal radiotherapy.^37^ This image registration and deformation strategy could differentiate between tumor progression and treatment-related abnormalities by looking at growth directionality. The harmonization and quantification of imaging methods and tumor growth may aid further development of artificial intelligence and machine learning programs in neuroradiology, which is currently being developed for various different applications and could aid in improving the diagnostic performance of MRI.^38^

## Conclusions

A clear correlation was found between the change in contrast-enhancing tumor in individual MRI data and white matter microstructure from the DTI template using the image registration and deformation strategy. Future studies with patient-specific DTI data are required to verify the accuracy and applicability of this method as a potential clinical metric for GBM patients in the pre and posttreatment setting.
